# Predicting long-term time to cardiovascular incidents using myocardial perfusion imaging and deep convolutional neural networks

**DOI:** 10.1038/s41598-024-54139-0

**Published:** 2024-02-15

**Authors:** Yi-Lian Li, Hsin-Bang Leu, Chien-Hsin Ting, Su-Shen Lim, Tsung-Ying Tsai, Cheng-Hsueh Wu, I-Fang Chung, Kung-Hao Liang

**Affiliations:** 1 Institute of Biomedical Informatics, National Yang Ming Chiao Tung University, Taipei City, Taiwan; 2 Department of Medicine, Taipei Veterans General Hospital, Taipei City, Taiwan; 3 Department of Nuclear Medicine, Taipei Veterans General Hospital, Taipei City, Taiwan; 4 Department of Medical Research, Taipei Veterans General Hospital, Taipei City, Taiwan

**Keywords:** End-to-end survival training, Survival analysis, Risk score, Multiresolution, Cardiology, Medical research

## Abstract

Myocardial perfusion imaging (MPI) is a clinical tool which can assess the heart's perfusion status, thereby revealing impairments in patients' cardiac function. Within the MPI modality, the acquired three-dimensional signals are typically represented as a sequence of two-dimensional grayscale tomographic images. Here, we proposed an end-to-end survival training approach for processing gray-scale MPI tomograms to generate a risk score which reflects subsequent time to cardiovascular incidents, including cardiovascular death, non-fatal myocardial infarction, and non-fatal ischemic stroke (collectively known as Major Adverse Cardiovascular Events; MACE) as well as Congestive Heart Failure (CHF). We recruited a total of 1928 patients who had undergone MPI followed by coronary interventions. Among them, 80% (n = 1540) were randomly reserved for the training and 5- fold cross-validation stage, while 20% (n = 388) were set aside for the testing stage. The end-to-end survival training can converge well in generating effective AI models via the fivefold cross-validation approach with 1540 patients. When a candidate model is evaluated using independent images, the model can stratify patients into below-median-risk (n = 194) and above-median-risk (n = 194) groups, the corresponding survival curves of the two groups have significant difference (P < 0.0001). We further stratify the above-median-risk group to the quartile 3 and 4 group (n = 97 each), and the three patient strata, referred to as the high, intermediate and low risk groups respectively, manifest statistically significant difference. Notably, the 5-year cardiovascular incident rate is less than 5% in the low-risk group (accounting for 50% of all patients), while the rate is nearly 40% in the high-risk group (accounting for 25% of all patients). Evaluation of patient subgroups revealed stronger effect size in patients with three blocked arteries (Hazard ratio [HR]: 18.377, 95% CI 3.719–90.801, p < 0.001), followed by those with two blocked vessels at HR 7.484 (95% CI 1.858–30.150; p = 0.005). Regarding stent placement, patients with a single stent displayed a HR of 4.410 (95% CI 1.399–13.904; p = 0.011). Patients with two stents show a HR of 10.699 (95% CI 2.262–50.601; p = 0.003), escalating notably to a HR of 57.446 (95% CI 1.922–1717.207; p = 0.019) for patients with three or more stents, indicating a substantial relationship between the disease severity and the predictive capability of the AI for subsequent cardiovascular inciidents. The success of the MPI AI model in stratifying patients into subgroups with distinct time-to-cardiovascular incidents demonstrated the feasibility of proposed end-to-end survival training approach.

## Introduction

Myocardial perfusion imaging (MPI) is a clinical modality which can be employed for assessing the cardiac perfusion in patients suspected of having compromised cardiac function^1–4^. These people may have occasional chest discomfort or shortness of breath^5^. After the examination, some may experience a slow and gradual disease progression, while others might see a more rapid deterioration in their cardiovascular (CV) functions^6^. Precise prediction of outcomes would be very valuable for the clinical management of the patients. The coronary arteries are essential to support oxygen to the myocardium. Individuals with impaired cardiac functions may exhibit constrained blood flow, particularly under stress conditions^7,8^. MPI detects and records blood flow via radioactive tracers and single-photon emission computed tomography (SPECT)^1^. Acquired three-dimensional (3D) MPI datasets are typically represented as a series of two-dimensional (2D) cross-sectional slices orthogonal to three mutually perpendicular axes (short, horizontal, and vertical long), traversing different depths within the heart^1^. This 2D tomographic representation can visualize cardiac conditions for human^9,10^. The MPI is a useful tool for facilitating the diagnosis of heart function impairments^1^, as well as for medical professionals to qualitatively foresee the future occurrence of major cardiovascular events.

In the realm of medical image processing such as color fundoscopy, X-rays, magnetic resonance imaging, computed tomography, ultrasound scans and MPI, the traditional workflow involves the sequential processing of images in two fundamental stages: (1) feature derivation, where specific attributes or patterns within the images are discerned and quantified. These attributes may encompass edge detection, texture analysis, or shape descriptors; (2) feature-value correlation with relevant clinical characteristics, such as disease diagnosis, staging, or treatment planning^11^. Notably, these conventional approaches necessitate the design of features, demanding domain expertise of human. Moreover, manual selection of the most informative features can be inherently challenging, potentially missing subtle or intricate patterns crucial for accurate diagnosis. The recent breakthroughs in artificial intelligence (AI), particularly deep neural networks, enabled streamlined end-to-end training which ushered in a paradigm shift. These networks mimic the structural organization of biological neurons and offer well-suited frameworks for addressing tasks such as computer vision, natural language processing, and, increasingly, medical image analysis. This deep learning approach signifies a departure from traditional methods where feature derivation is done by manual, labor-intensive efforts. Among the diverse deep neural network architectures, the convolutional neural network (CNN) is particularly suitable to extracting spatial features^12^. The convolutional kernels (also known as filters) can extract localized spatial features at multiple scales, a capability particularly apt for processing images^13^. Furthermore, in typical CNN architectures, the extracted features undergo aggregation, facilitated by max pooling or average pooling functions. The convolutional layers and pooling layers are interwoven within the network's architecture, resulting in a pyramidal, encoder structure, wherein contextual information is progressively distilled, layer by layer. In the applications of deep learning technique to MPI, CNN has demonstrated superior performance compared with multi-layer perceptron in classification tasks^14^.

Nie *et.al.* used CNN to process magnetic resonance imaging (MRI) and derive features, which were sent to a support vector machine (SVM) module for patient classification^15^. The two-step procedure does not function as a comprehensive end-to-end solution. Berkaya et al*.* developed a CNN and SVM based classification models to classify normal and abnormal (ischemia or infarction) SPECT MPI, where the CNN was used for the feature derivation, and SVM was subsequently used for the classification, i.e., a two-step process^16^. Papandrianos et al. also addressed the classification problem, aiming to diagnose SPECT MPI images and achieve automatic classification into normal or ischemic categories^17^. The model utilized VGG-16 and DenseNet-121 pre-trained networks to obtain optimal results. Liu et al. examined the idea of using stress-state MPI images alone to automatically classify normal images and those with myocardial perfusion abnormalities^18^. The performance of the AI model was similar to that of the conventional quantitative defect size method^18^. Zahiri et al*.*^19^ and Apostolopoulos et al.^20^ again aimed at classifying disease and non-disease patients. However, they employed a different approach in that they did not directly analyze the 2-D tomograms. Instead, they used a semi-manually processed formats called polar maps, which are of a circular visualization format showing the distribution of blood flow for the ease of human perception. Polar maps are derived from tomograms yet the procedure is not standardized across medical institutes which may hinder the wide use of this approach in many institutes.

Survival analysis is an important method in medical research for the assessment of time-to-event outcomes. It focuses on time-dependent event data, providing crucial insights into patient outcomes, treatment efficacy, and long-term prognosis, which cannot be adequately addressed by the binary classification of disease and non-disease. One important aspect of survival analysis is that it can handle the condition that some individuals have not experience the event during the follow-up period, referred to as "censored" data points, making it more flexible and appropriate for real-world medical data. Survival analysis provides a method of analyzing time-dependent outcomes and allows researchers to assess not only the presence or absence of disease but also the timing and duration of events. With respect to the AI models for survival analysis, Zhu et al. proposed the Whole Slide Histopathological Images Survival Analysis framework (WSISA) to extract features from pathological slide images, finding discriminative patches and then predict patient survival status^21^. Tang et al. utilized Capsule network to process the whole slide histopathological images to estimate the survival rates of glioblastoma and lung squamous cell carcinoma^22^. However, both the two teams have not visualized longitudinal survival curves in the testing dataset.

Although CNN has been widely used for classification problems, a significant unmet need remains in the use of end-to-end deep learning technology directly for survival analysis. Therefore, in this study, we proposed a novel end-to-end survival training approach, and used a stringent cross-validation and testing procedure to demonstrate the effectiveness of this approach in predicting time to cardiovascular incidents after the MPI examination.

## Methods

### The patient cohort

**Figure 1:**
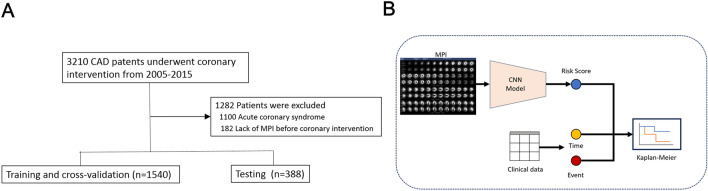
Schematic diagrams of this research. (**A**) The flowchart of patient selection for this study. (**B**) Schematic diagram of the proposed end-to-end survival training architecture designed to evaluate the risk of patients with respect to their subsequent time to cardiovascular incidents. Myocardial perfusion images presented as a series of two-dimensional gray-scale slices which are perpendicular to the short, long-vertical and long-horizontal axes of the heart. This 2D tomographic presentation is defined as the input format of our MPI AI model, trained end-to-end with optimization functions related to the survival analysis. The risk scores derived from MPI AI model reflect patients’ subsequent outcome. Patient strata by the risk score can be analyzed using Kaplan–Meier plots.

A retrospective cohort of 3118 patients with symptomatic Coronary Artery Disease (CAD) who received coronary intervention at the Taipei Veterans General Hospital between 2005 and 2015 were screened. Successful coronary interventions were performed and patients were followed in our outpatient clinic. This cohort was a retrospective observational study that complied with the Declaration of Helsinki and was approved by the appropriate Health Authorities, Independent Ethics Committees, and Independent Review Boards in Taipei Veterans General Hospital (2016-03-014CC). This study aimed to generate an MPI-AI model for subsequent outcome prediction in stable patients who received coronary intervention, therefore, patients who received coronary intervention due to acute coronary syndrome (myocardial infarction or unstable angina) or did not have MPI taken before coronary intervention were excluded for this analysis. This results in a cohort of 1928 moderate-to-severe ischemia patients for this study (Fig. 1A). All patients included in this study have given informed consents. After successful coronary intervention, patients of this study were treated following the AHA guidelines for clinical practice and were followed regularly^23^. The study examined the feasibility of end-to-end survival training, where the SPECT MPI scans are used to train the network to generate risk scores reflecting the subsequent cardiovascular events of interests (Fig. 1B). MPI taken before coronary intervention were obtained retrospectively from the Picture Archiving and Communication System (PACS) of the hospital.

### Study outcomes and clinical events

The primary outcome was the composite of non-fatal myocardial infarction, non-fatal ischemic stroke, cardiovascular death (collectively referred to as Major Adverse Cardiovascular Events; MACE) and hospitalization for congestive heart failure (CHF). All these events together were referred to as the Total CV Events. Myocardial infarction was confirmed in patients presenting with ischemic symptoms with elevated serum cardiac enzyme levels and/or characteristic electrocardiogram (ECG) changes. Ischemic stroke was confirmed as an obstruction within a blood vessel supplying blood to the brain with imaging evidence by either MRI or CT scan and new neurological deficit lasting for at least 24 hours. The protocol for CV event follow-up was performed as previously described^24,25^. We investigate the timing of occurrence of these cardiovascular incidents and the timing of MPI examinations, calculating the time to events. We also study clinical variables such as age, sex presence of diabetes, hypertension, hyperlipidemia, and smoking habits.

The MPI AI model needs to perform well on new data not specifically trained on, i.e., the capability of generalization. A model with such capability implied that it has learned the underlying patterns and relationships in the data rather than just memorizing the training data. To achieve this, the patients were randomly assigned into training/cross-validation cohort and testing cohort at the proportions of 80% and 20%, without one single patient appears in both cohorts (Fig. 1A). The model was derived from a training/cross-validation cohort and evaluated in an independent testing cohort.

### End-to-end survival training for estimating the risks of cardiovascular incidents

We proposed here an end-to-end survival training approach, which aims to estimate the time to events using the baseline medical images. A specific task was used to demonstrate the feasibility of this approach, that is, training a MPI AI model for assessing patients' risk with respect to future cardiovascular incidents and stratifying patients for Kaplan–Meier plot analysis (Fig. 1B). The events of interest are the total cardiovascular events (including MACE and CHF) of patients, and the input image is MPI scan presented in a format of 2D tomogram. This format comprises a total of 96 slices, including 48 slices taken at rest condition and 48 at stress condition. Each condition has 24 slices taken perpendicular to the short axis, 12 slices perpendicular to the horizontal long axis and 12 slices perpendicular to the vertical long axis. We used the CNN architecture, particularly ResNet50, for implementing the MPI AI model. The outstanding feature of ResNet lies in its use of skip connections or residual connections, addressing the vanishing gradient problem encountered in deep networks. Its effectiveness and stability have been extensively validated in numerous studies^26–28^.

In the end-to-end survival training, we employed a stochastic gradient ascent method with MPIs in batches used for the training. The main difference between the proposed end-to-end survival training and conventional CNN is the optimization function. In survival analysis, some individuals did not experience the cardiovascular incidents during the study period before the end of follow-up, i.e. the "censored" observations. The Cox proportional hazards model, also known as the Cox regression, is often used for survival analysis where the goal is to maximize the likelihood function by choosing adequate coefficients of covariates (independent variables) in the regression equation. The likelihood function properly accounts for censored data, ensuring that the contribution of each individual is appropriately weighted.

 In end-to-end deep learning, we incorporated major concepts from Cox regression which captures the relationship between the survival time of subjects and predictor variables (in this case, the MPI in the format of numerical matrices, denoted as x). Data on the survival time (time to an event) are used for modeling the effect of predictor variables (x) on the hazard function of cardiovascular incidents using the following equation:

1 $$ {\text{h}}\left({{\text{t}}|{\text{ x}}_{\text{i}}} \right) \, = {\text{ h}}_{0} \left({\text{t}} \right){\text{e}}^{{{\text{f}}\left( {{\text{x}}_{\text{i}}} \right)}} ,$$

where *h*(*t|x*_*i*_) is the time (*t*)-dependent hazard function for an individual *i* with specific values of predictors *x*_i_. *h*_*0*_(*t*) is the baseline hazard at time *t*. *x*_i_ represents the input of the MPI model from  patient *i*. *f*(*x*_i_) is the risk score generated by the MPI AI survival model estimated for the predictor variable *x*_i_. During the training and validation stage, we estimate *f*(*x*) using the partial likelihood as the optimization function. In each batch of the training process, the patient’s log likelihood is calculated:

2 $$log\left(L\right)=\sum_{i}{\delta }_{i}\left[f\left({x}_{i}\right)-log\left(\sum_{j\in R\left({t}_{i}\right)}exp\left(f\left({x}_{j}\right)\right)\right)\right],$$

where *δ*_i_ is the event (cardiovascular incident) indicator for patients from whom the image was taken. *δ*_i _= 0 represents no event, and 1 represents an event. *R*(*t*_i_) represents the set of all patients for whom patient *j*’s survival time is equal to or greater than the time point *t*_i_.

In the study, we conducted an end-to-end survival training on the training and cross-validation cohort, where the model is referred to as the MPI AI model. The output of MPI AI is a risk score which should reflect the time to major cardiovascular events given the MPI scan as inputs. The evaluation metrics for our model performance included the C-index and statistical significance of log-rank tests. The fivefold cross-validation procedure was used to generate models in the training/cross-validation cohort. We utilized the validation set model with the highest C-index among the 1000, 2000, 3000 day analysis as the predictive model for the testing dataset.

We conducted experiments on a server with an NVIDIA A6000 GPU, operating under the Ubuntu 20.04 operating system. All model training and evaluation were carried out using the Python 3.8.11 programming language and the PyTorch 1.12.1 framework. The hyperparameters used for the AI model construction is shown in Supplementary Table 1^29^. Parameter configuration aimed to avoid image size reduction and minimize information loss, using the original image size as input and setting a batch size of 16 to optimize GPU memory and training speed. Adam optimizer was used to provide adaptive learning for network coefficients, preventing rapid convergence and potential overfitting with a relatively low learning rate of 0.0001. The epoch number was chosen as 70 which is sufficient to perform a successfully training. These parameter settings demonstrated stable outcomes in both training and testing sets during fivefold validation, indicating consistent results.

### Performance evaluation

Performance of the MPI AI with respect to patient stratification were evaluated using Kaplan–Meier plots and Receiver Operating Characteristic (ROC) curves. Kaplan–Meier plots were employed to analyze survival probabilities, providing insights into event occurrences over time for different risk groups. As the cardiovascular incidents occur in different times after baseline, we use ROC for classifying patients at different time points (such as 1000, 2000 and 3000 days after baseline), or with or without cardiovascular events, disregarding event timing or censoring. ROC curves were also used when patients have been stratified into low, intermediate, and high-risk groups. In such cases, ROC curves are composed of three straight lines connected by two turning points, where the two turning points indicate the sensitivity and specificity when the median risk score and the score that separate quartiles 3 and 4 were used as thresholds.

## Results

### Patient characteristics

A total of 1,928 patients who had received MPI examinations and coronary intervention were analyzed. The clinical characteristics of the training and cross-validation cohort and testing cohort were compared, and no significant difference of the values of basic clinical variables between the two cohorts were found (Table 1).

**Table 1:** Baseline characteristics of study population.

	Training and cross-validation	Testing	p
	N = 1540	N = 388	
Age	69.12 ± 12.43	68.45 ± 12.65	0.343
Male, n (%)	1117 (72.53)	295 (76.03)	0.164
BMI, Kg/m^2^	25.70 ± 4.07	25.93 ± 3.31	0.254
Hypertension, n (%)	1022 (66.36)	248 (63.92)	0.364
Diabetes, n (%)	558 (36.23)	150 (38.66)	0.376
Systolic BP, mmHg	131.84 ± 20.29	132.08 ± 20.34	0.838
Diastolic BP, mmHg	74.04 ± 11.49	73.97 ± 12.52	0.926
LVEF, %	54.24 ± 12.03	53.84 ± 10.65	0.721
Glucose, mg/dL	121.28 ± 41.65	122.83 ± 41.92	0.569
Cholesterol, mg/dL	173.83 ± 42.01	174.16 ± 40.29	0.892
LDL-C, mg/dL	106.73 ± 34.63	108.11 ± 33.71	0.488
HDL-C, mg/dL	43.32 ± 12.36	43.13 ± 11.02	0.770
Triglyceride, mg/dL	136.74 ± 83.24	137.03 ± 88.30	0.953
TC/HDL-C ratio	4.28 ± 1.52	4.26 ± 1.43	0.809
Creatinine	1.54 ± 1.68	1.52 ± 1.72	0.890
CAD severity			
SVD, n (%)	106 (27.32)	438 (28.44)	0.701
DVD, n (%)	126 (32.47)	507 (32.92)	
TVD, n (%)	155 (39.95)	594 (38.57)	
Total stents, n	1.85 ± 1.08	1.78 ± 1.01	0.295
Total stents size, mm	3.06 ± 0.44	3.05 ± 0.40	0.670
Total stents length, mm	23.36 ± 5.99	23.10 ± 6.27	0.494
Medication			
ACE inhibitor/ARB, n (%)	842 (54.68)	210 (54.12)	0.845
β-blocker, n(%)	787 (51.10)	199 (51.29)	0.948
Calcium channel blocker, n (%)	586 (38.05)	163 (42.01)	0.153
Diuretics, n (%)	194 (12.60)	54 (13.92)	0.488
Statin, n (%)	897 (58.25)	219 (56.44)	0.520

Data are mean ± SD, BMI indicates body mass index.

*BP*, blood pressure, *LDL-C* low density lipoprotein-cholesterol, *HDL* high density lipoprotein-cholesterol, *CAD* coronary artery disease, *SVD* single vessel disease, *DVD* double vessel disease, *TVD* triple vessel disease, *ACE* angiotensin converting enzyme, *ARB* angiotensinogen receptor blocker.

### Total CV events during follow-up

Clinical follow-up was carried out with all patients for a mean period of 1789 ± 983 days. During this time, there was 58 (14.95%) total CV events in the testing cohort and 228 (14.81%) total CV events in training and cross-validation cohort identified. All events were presented in Table 2 and the event rate was similar between two groups (Table 2).

**Table 2:** Clinical adverse cardiovascular events.

	Training and cross-validation	Testing	p
	N = 1540	N = 388	
Non-fatal MI	72 (4.68)	19 (4.90)	0.854
Stroke	23 (1.49)	8 (2.06)	0.426
CV death	33 (2.14)	5 (1.29)	0.279
MACE	119 (7.73)	32 (8.25)	0.733
CHF hospitalization	128 (8.31)	35 (9.02)	0.654
All CV event	228 (14.81)	58 (14.95)	0.943

*MACE* major adverse cardiovascular event.

All CV events include MACE and CHF hospitalization.

### MPI AI model generation

**Figure 2:**
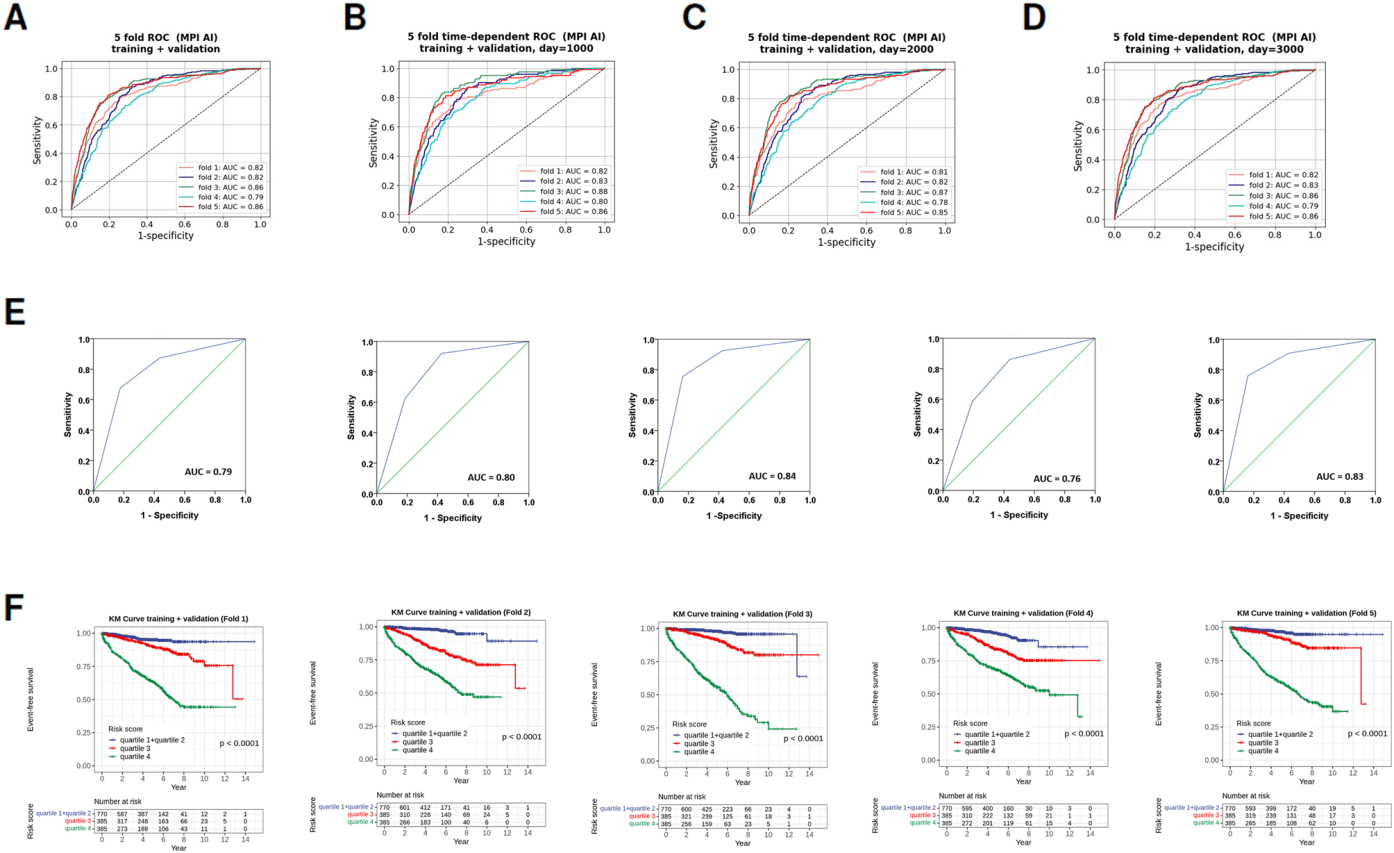
MPI AI model performance in the training/cross-validation stage (n = 1540). (**A**) The receiver operating characteristic curves (ROC) of 5 models generated in the fivefold analysis, for classifying patients with or without events regardless of the time of event occurrence. (**B**) The ROC of detecting events occurred within the first 1000 days of observation. (**C**) The ROC of detecting events occurred within the first 2000 days of observation. (**D**) The ROC of detecting events occurred within the first 3000 days of observation. (**E**) The ROCs after the patients are divided into three subgroups with different risk levels, using the median risk score and the threshold that separate quartiles 3 and 4. (**F**) The Kaplan–Meier plots of patient strata, separated into three different risk subgroups.

MPI AI model were trained using the baseline MPI of the training/cross-validation cohort (n = 1540), and the performance is presented in Fig. 2. The ROCs of models for classifying patients with or without events regardless of the time of event occurrence, or at 1000, 2000 and 3000 days, are presented in Fig. 2A–D respectively. The study employed a fivefold validation process, hence these figure panels depict five ROC curves, each corresponding to a different segmentation of training and validation data. The five ROCs for classifying patients with or without events regardless of the time of event occurrence, when patients are categorized as three different risk subgroups (i.e. quartile 1 and 2 combined, quartile 3 and quartile 4) are shown in Fig. 2E. These ROCs, composed of three straight lines with two turning points, present risk stratification into low, intermediate, and high-risk groups using different thresholds. The Kaplan–Meier plot of the three risk subgroups of patients is shown in Fig. 2F. The model performances across folds are relatively robust, as the curves in Fig. 2A–D are relatively close to each other.

**Figure 3:**
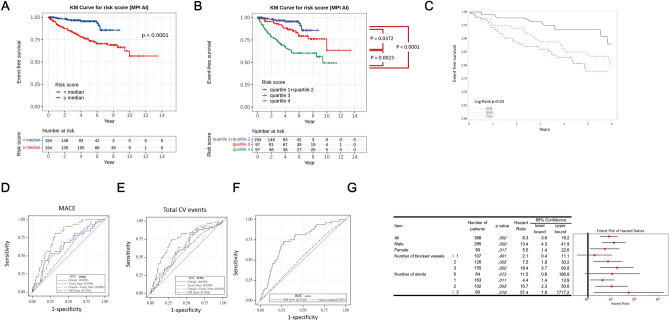
MPI AI model performance in the testing cohort (n = 388). (**A**) The risk score derived from the baseline MPI images can stratify patients into below-median-risk group (shown in blue color, also referred to as the low-risk group) and above median-risk group (shown in red color), which have significant difference in their time to cardiovascular incidents (P < 0.0001). (**B**) The above-median risk group were further stratified into two equal-sized groups (n = 97 each), and referred to as the high (shown in green color, and corresponds to the 4th quartile) and intermediate (shown in red color, and corresponds to the 3rd quartile) risk groups respectively. The corresponding survival curves of the two groups have significant difference (log-rank P = 0.0023). The intermediate risk and the low risk groups, also manifest statistically significant difference (log-rank P = 0.0472). When the low-risk group (corresponds to the 1st and 2nd quartiles together, accounts for 50% of all patients) and the high-risk group (accounts for 25% of all patients) are compared, the difference in survival curves is very significant (log-rank P < 0.0001). (**C**) The Kaplan–Meier plots of patients stratified as single vessel disease (SVD), double vessel disease (DVD) and thriple vessel disease (TVD). (**D**) The ROC of clinical model, vessel number, clinical and vessel combined model, as well as the MPI score for MACE events. (**E**) The ROC for Total CV events. (**F**) The ROC of patients stratified using the stent number. (**G**) The forest plot illustrates hazard ratios (HR) and their 95% confidence intervals (CI) for various clinical and ICA factors on the association between MPI derived scores and cardiovascular incidents. Among 388 patients, the score showed a strong association (HR: 8.328, 95% CI 3.604–19.245; p < 0.001). Male patients (n = 295) exhibited a higher association (HR: 13.411, 95% CI 4.290–41.926; p < 0.001) compared to the overall population, while females (n = 93) displayed a significant but comparatively lower association (HR: 5.535, 95% CI 1.355–22.603; p = 0.017). Evaluation of blocked vessels revealed stronger associations in patients with more blockages (HR: 18.377 for three vessels, p < 0.001), while the presence of stents showed a rising trend in model performance, peaking notably in cases with three or more stents (HR: 57.446; p = 0.019) despite a smaller sample size.

We then employ the candidate model to stratify patients of the testing cohort, an independent cohort from the training/validation cohort, using the MPI AI model. Patients in the testing cohort are stratified into two distinct risk groups: those with a risk score below the median and those with a risk score above the median. The distinct patterns of the patient strata in their time to major adverse cardiovascular events are graphically represented as Kaplan–Meier plots, and their significant difference are evaluated using log-rank tests (P < 0.0001), indicating that the MPI AI-derived risk score is a robust predictor of major adverse cardiovascular events (Fig. 3A). Within the above-median risk group, we performed additional stratification, dividing it into two subgroups: quartile 3 and quartile 4, and referred to as the intermediate risk group and high-risk group respectively, for evaluating whether the AI model can further stratify patients with different levels of risks. The three strata revealed visually different survival curves in the Kaplan Meier plot (Fig. 3B). The high and intermediate risk groups have significant difference (P = 0.0023). The intermediate-risk and the low-risk groups also manifest statistically significant difference (P = 0.0472). When the low-risk group (accounting for 50% of all patients) and the high-risk group (accounting for 25% of all patients) are compared, the difference in survival curves is very significant (P < 0.0001). Notably, the 5-year cardiovascular incident rate is less than 5% in the low-risk group, while the rate is nearly 40% in the high-risk group.

We further evaluated the number of blocked vessels, assessed by the interventional cardiologists regarding the three major coronary arteries—Left Anterior Descending (LAD), Left Circumflex (LCx), and Right Coronary Artery (RCA). The grading of blockages established severity levels, further stratifying this moderate-to-severe patient population with their different risks: single vessel disease (SVD), double vessel disease (DVD), and triple vessel disease (TVD). In Fig. 3C, the Kaplan–Meier plots exhibit the survival analysis of patients categorized into three groups based on their coronary artery disease severity. It showed the survival curve of the occurrence of total CV events depends on the diseased vessel number. The risk of developing total CV events correlated with underling coronary disease severity. To evaluate the value of MPI-AI derived risk score in clinical use, its performance was compared with the uses of coronary artery severity. In addition, traditional risk factors including age, gender, history of hypertension and diabetes were also compared. For MACE, the AUC of MPI-AI risk score is 0.779, which is significant higher than that of the clinical risk factors (AUC: 0.639); disease vessel numbers (AUC:0.577); combined traditional risk factors and disease vessels (AUC:0.647) (Fig. 3D) For All CV events, the AUC of MPI-AI risk score is 0.747, the clinical risk factors (AUC: 0.620); disease vessel numbers (AUC:0.577) and AUC of combined traditional risk factors and disease vessels is 0.636 (Fig. 3E; Table 3). Notably, the model, trained solely on total CV events, exhibits strong predictive performance for MACE as well. MPI-AI derived risk score have significant greater improvement in future outcome prediction than considering of clinical traditional risk factors and underlying coronary disease severity. In addition, AUC of the MPI-AI risk (AUC:0.747) is significantly higher than stent number deployed (0.530), indicating MPI-AI derived risk score provide better predictive value than stenosis lesions in clinical practice (Fig. 3F). Figure 3D–F revealed the limitations of using conventional classifiers such as blocked vessel or stent numbers and the excellency of MPI derived score in predicting imminent MACE or total CV events.

**Table 3:** Comparison of predictors for future events.

	AUC	P
MACE		
Clinical traditional risk factors	0.651 (0.559–0.743)	0.060
Disease vessels numbers	0.557 (0.459–0.655)	0.304
Combined traditional risk + Disease vessel number	0.653 (0.561–0.745)^※^	0.094
MPI-AI risk score	0.727 (0.634–0.821)*^Ψ※^	< 0.0001
Total CV events		
Clinical traditional risk factors	0.620 (0.544–0.695)	0.098
Disease vessels numbers	0.577 (0.504–0.650)	0.058
Combined traditional risk + Disease vessel number	0.636 (0.562–0.711)*^※^	0.075
MPI-AI risk score	0.747 (0.679–0.814)*^Ψ※^	< 0.0001

Clinical risk factors included age, gender, history of hypertension and diabetes.

*Indicates P < 0.05 compared with clinical risk factors.

^Ψ^Indicates p < 0.05 compared with combined traditional risk factors + disease vessel numbers.

^※^Indicates p < 0.05 compared with disease vessel numbers.

The subgroup analysis of the MPI AI score showed that patients with three blocked vessels (n = 155) exhibit a substantially higher hazard ratio of 18.377 (95% CI 3.719–90.801; p < 0.001), followed by the subgroup with two blocked vessels (HR = 7.484, 95% CI 1.858–30.150; p = 0.005) and the subgroup with blocked vessel number ≤ 1 (HR = 2.060, no statistical significance). This suggests that the MPI AI model is suitable for patients with blocked vessel number ≥ 2 (Fig. 3G). We also evaluated the subgroups with different number of stent placement. Patients without stents (n = 64) display a hazard ratio of 11.535 (95% CI 0.797–166.933) but have not achieved statistical significance. Those with a single stent (n = 163) exhibit a hazard ratio of 4.410 (95% CI 1.399–13.904; p = 0.011). Patients with two stents (n = 102) show a hazard ratio of 10.699 (95% CI 2.262–50.601; p = 0.003). In the extreme cases where patients with three or more stents (≥ 3) (n = 60) show a remarkably high hazard ratio of 57.446 (95% CI 1.922–1717.207; p = 0.019), indicating a potentially substantial association between multiple stents and stenosis risk, despite the small sample size. Overall, the hazard ratio increases notably as the number of stents inserted rises.

### The performance of the end-to-end-derived MPI AI model is comparable with the model utilizing clinical variables

**Figure 4:**
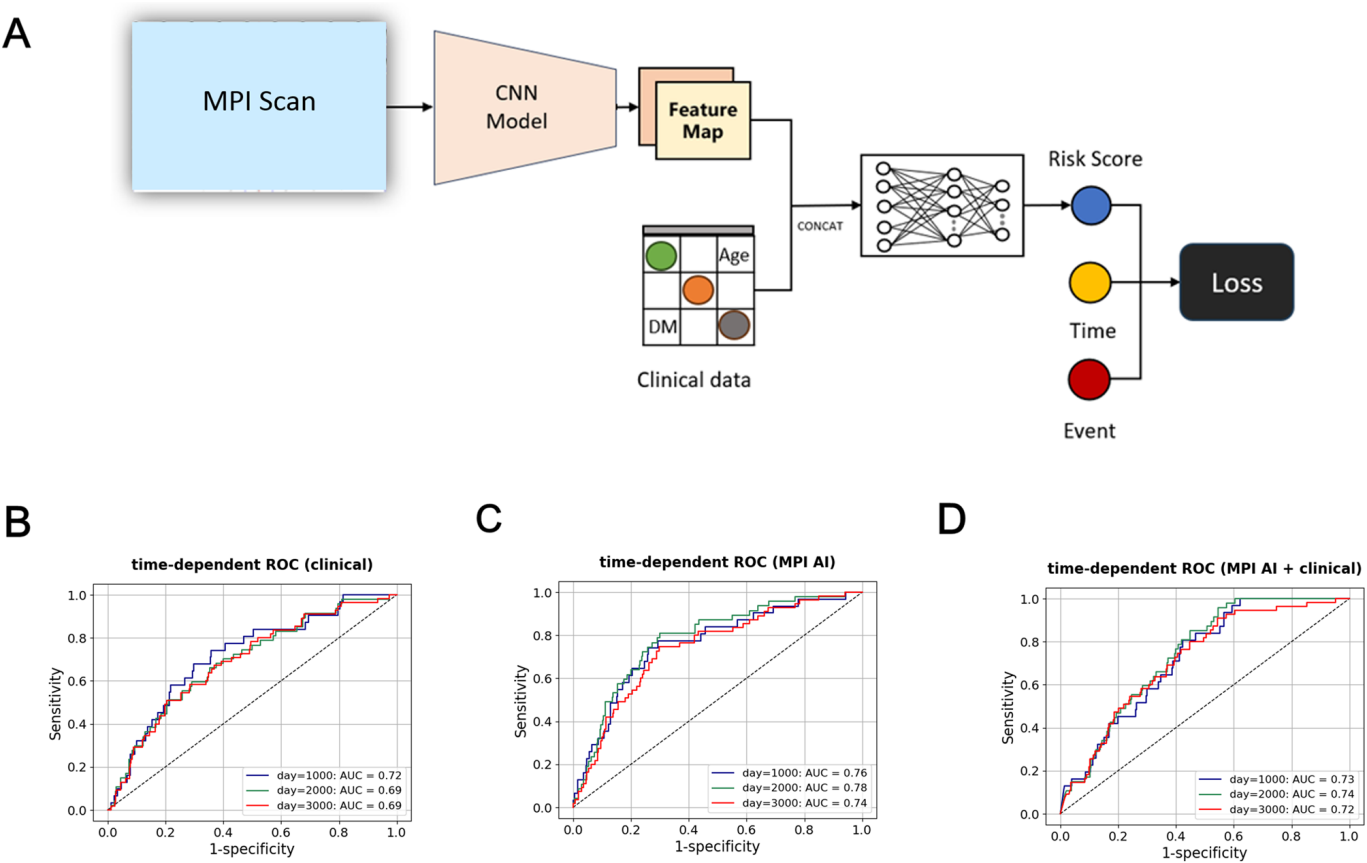
(**A**) This generalized schematic diagram accommodating inputs with the MPI only, with the clinical variable only, or with the combination of clinical variables and MPI. (**B**) The performance of the clinical AI model; (**C**) the MPI AI model, and (**D**) the combined model for classifying patients with or without cardiovascular incidents at 1000, 2000, and 3000 days after baseline, visualized as the time-dependent receiver operating characteristic curves.

We also evaluated whether the addition of clinical information into the neural network can further improve the performance. To do so, we designed a generalized framework to evaluate models with or without clinical data (Fig. 4A). For model evaluations, we introduced three different representative time points, i.e., 1000 days, 2000 days, and 3000 days after baseline. The performance of the multivariate clinical model is then shown as the time-dependent receiver operating characteristic curve at these time points. Heart diseases related clinical variables, including age, sex, diabetes, hypertension, hyperlipidemia, and smoking, are provided as inputs into the neural network. The performance of the clinical variable neural network in the independent testing dataset is shown in Fig. 4B, where the highest AUC was reached in classifying events before day 1000 (AUC = 0.72). For the MPI AI alone model, the highest AUC was reached in classifying events before day 2000 (AUC = 0.78, Fig. 4C). When the clinical data served as additional entry nodes to the MPI AI model, the highest AUC was reached in classifying events before day 2000 (AUC = 0.74, Fig. 4D). This demonstrates that using MPI images alone yielded the most favorable results.

## Discussions

In this study, we employed deep learning technology to autonomously discover intricate associations of a dense array of signals such as MPI with clinical events and times, revolutionizing survival analysis which was previously limited to the scope of finite number of clinical variables. This transformative shift has unleashed the potential for automated, hands-free extraction of image features which can adequately reflect patients’ outcome. The proposed novel approach, end-to-end survival training, was applied successfully to the risk assessment of cardiovascular incidents using MPI, which are processed by the AI model trained in one cohort and validated in an independent cohort with images unseen previously. The trained model is capable of stratifying patients in the testing cohort into risk groups. Stratification based on median risk scores and quartiles allows for the distinguishing of different risk groups. The study's strength lies in its ability to visually represent distinct patterns of patient strata in Kaplan–Meier plots, demonstrating clear differences in time to cardiovascular incidents. This demonstrates the robust predictive power of AI-derived risk scores based on baseline MPI images.

In the current clinical guidelines (e.g. 2021 AHA guidelines), patients with moderate-to-severe ischemia on MPI are recommended for ICA^23^. This research was conducted under the viewpoint that patients with moderate to severe ischemia are confronted by their varying, imminent risk of cardiovascular incidents. The estimation of risks for individuals in this population can facilitate their treatment planning. The patients included into this study were all considered to have moderate-to-severe conditions, and had received ICA as well as MPI. The risk stratification using baseline MPI demonstrated significant implications for cardiovascular risk assessment. We want to set aside approximately one quarter of patients that has a particularly high elevated risk of subsequent cardiovascular incidents who might benefit most from closer monitoring. Stratifying patients into low and high-risk groups based on median risk scores provides a straightforward categorization, aiding in comparative analysis. It allows for a clear differentiation between patients with lower versus higher predicted risks. Dividing the high-risk group further into quartiles allows for a more nuanced analysis within this cohort. Quartiles 3 and 4 represent subsets of patients with progressively higher risk scores within the high-risk category, enabling the identification of even higher-risk subgroups. In this moderate-to-severe patient population, the 5-year cardiovascular incident rate is less than 5% in the low-risk group identified by our AI model (accounting for 50% of all patients), while the rate is nearly 40% in the high-risk group (accounting for 25% of all patients), who should receive more attention due to their elevated risk.

MPI is common used tool for CAD diagnosis which can provide the information of target diseased vessel and ischemic myocardium involved. Our study first generated a MPI-AI algorithm for future outcome prediction in CAD patients after successful coronary intervention. To our interest, the risk score from MPI-AI algorithm has significant predictive values than number of disease vessels or stents implanted, suggesting this risk score from MPI-AI algorithm could be used as risk stratification in stable CAD patients after successful PCI. In Fig. 3D and E, we showed that the number of coronary artery blockages alone does not reflect the risk of MACE/Total cardiovascular incidents as good as our MPI AI model.

We also evaluated the model performance on a predictive score for cardiovascular incidents in various clinical subgroups (Fig. 3G). The forest plot demonstrates how different factors such as gender, the number of blocked vessels, and stent placement impact the risk of stenosis, providing insights into the varying degrees of association given these clinical and angiography factors. Among the patients in the testing cohort, the score showed a strong association. Male patients exhibited a higher association compared to the overall population, while females (n = 93) displayed a significant but comparatively lower association. Evaluation of blocked vessels revealed stronger associations in patients with more blockages, while increased stent numbers correlated with higher hazard ratios for stenosis risk. In the moderate-to-severe patient group, the response to treatment and disease progression can differ significantly even for patients with the same number of blockage (2 or 3), or receiving the same number of stent (1 or 2), an observation which indicates the importance of using MPI AI for fine-grain stratification. Although some of the subgroups does not show statistical significance due to reduced sample size, the general trend in the forest plot showed that hazard ratio increases as the disease severity increases, a trend that is consistent with the intuition and suggests that the AI model has captured the essence of the disease severity. Factors such as the presence and effectiveness of collateral circulation around blocked arteries can vary between individuals. This collateral circulation might mitigate the impact of severe blockages in some patients, reducing their imminent risk. Furthermore, some plaques might be stable, while others are prone to rupture, leading to a sudden blockage and subsequent cardiovascular incidents. Imaging techniques like intravascular ultrasound (IVUS) or optical coherence tomography (OCT) may provide insights into plaque characteristics, helping predict potential risks, after these new modalities achieve wide acceptance and be introduced to the clinical work^30,31^.

The time-dependent receiver operating characteristic curves, as illustrated in Fig. 4B–D, provide insights into these analyses. It is evident from the figures that the clinical model excels in predicting cardiovascular incident events occurring within the first 1000 days (as indicated by the ROC curve being closest to the outermost point), while the MPI AI model performs optimally in predicting events occurring within 2000 days. The combination of both models yields the best prediction performance for cardiovascular incident events occurring within 2000 days. In other words, the clinical model is more suitable for more imminent events, while the MPI AI model is capable of predicting long-term events. While clinical variables are relevant to heart diseases, we found that their incorporation alongside MPI might not always enhance predictive performance. While this may seem beneficial by incorporating more information, the addition of clinical variables to the MPI-AI neural network could introduce increased complexity. Interactions between the MPI-AI features and clinical variables might not be adequately captured or might introduce unexpected interactions that diminish the model's predictive power. Failure to account for these complex interactions could impact the model's accuracy. Additionally, the neural network might inherently prioritize certain types of data over others. For example, the model might be biased towards learning more from clinical variables rather than the MPI. This bias could overshadow the potential synergistic contribution of clinical information and MPI, leading to relatively poorer performance. Furthermore, the timing of when the clinical variables are most informative might differ from the timing of the MPI in predicting events. As seen in the results, the highest AUC for the clinical model was before day 1000, whereas the MPI images alone showed better performance before day 2000. This discrepancy suggests that the clinical variables might not offer additional relevant information within the specific timeframes being assessed. While our initial attempt to include clinical variables alongside MPI didn't yield improved results, refining this approach could involve feature selection, identifying more informative clinical variables, or modifying the way these variables are integrated into the model.

The end-to-end survival training approach can be applied to coronary Computed Tomography Angiography (CTA). This requires the use CTA as the input signal for training the AI model. The loss function is the same as the MPI AI model. The use of CTA is a good direction for our future research. Rather than utilizing individual patient risk scores as the ground truth, our methodology directly employed the time-to-event data, comprising both the occurrence and timing of censored and non-censored events, within our end-to-end training process. This approach aligns with the principles of Cox regression, aiming to ascertain coefficients for a regression equation that elucidates the time-to-event dynamics within a patient batch.

The MPI AI model, although powerful, still have inherent limitations. We did not include negative-mild ischemia patients in the current study, which makes the current AI model incapable of estimating the risk of patients who still have varying degrees of cardiovascular risks despite not showing perfusion abnormalities. Patients with negative or mildly ischemic MPI results could still benefit from preventive strategies to mitigate potential future risks, in this sense, the current AI model has yet to provide a complete picture of the entire patient spectrum. Therefore, we will incorporate negative-mild ischemia patient populations into our future study, offering opportunities to these patients for early intervention and tailored management plans.

In the future, we plan to do the following to potentially improve the model's performance. We will evaluate model with different scales such as ResNet18 and ResNet101 in additional to Res50 for a more comprehensive assessment. Increasing the sample size of the training dataset in the current study is crucial for improving the model's performance. We will employ techniques such as semi-supervised learning to utilize unlabeled data in conjunction with the limited labeled dataset. These methods can leverage the structure within the data to improve model performance even with limited labeled dataset. Additionally, Combining datasets from multiple sources can potentially compensate for the limitations of a small dataset and enhance model performance. We will seek collaborations with other institutions or research groups, probably with the federated learning technology, to access larger datasets for validation or fine-tuning purposes. Furthermore, we will use stopping criteria to dynamically determine the length of training/validation, as opposed to the current method of fixing the number of epochs for training. We will also improve model interpretability using techniques like saliency maps or GradCAM visualization^29^ which can provide insights into the features or areas that is crucial for reflecting subsequent risks of cardiovascular incidents, aiding clinicians in the interpretation of MPI.

## Conclusion

We demonstrated feasibility of the proposed end-to-end survival training for patient stratification according to the estimated risk of subsequent cardiovascular incidents. By leveraging this technology, the research sought to maximize the power of deep learning with the learned features from MPI which can be used to indicate cardiovascular incidents. These findings underscore the potential clinical utility of the trained MPI AI model in future risk assessment and highlight the ability to further refine risk stratification within high-risk patient populations, such as those who have three blocked vessels. The trained model has significant implications for patient management and the development of personalized healthcare strategies in the context of cardiovascular disease.

### Supplementary Information

Supplementary Table 1.

## Data Availability

The datasets generated and/or analyzed during the current study are not publicly available, as we are still investigating a suitable approach of data release. Currently, data will be available for academic scientists upon reasonable request to the corresponding authors.
