# Supplementary material for: Predicting long-term time to cardiovascular incidents using myocardial perfusion imaging and deep convolutional neural networks
Source: Sci Rep. 2024 Feb 15;14:3802. doi: 10.1038/s41598-024-54139-0 (PMC10869727; doi:10.1038/s41598-024-54139-0)
Supplement: Supplementary file 1 — Supplementary Table 1. [file 41598_2024_54139_MOESM1_ESM.docx]

**Supplementary Table 1**. The hyperparameters for the CNN and the training procedure

| Hyperparameters | Value |
| --- | --- |
| Image size | 668$\times$943 |
| Images in a batch | 16 |
| Epoch | 70 |
| Optimizer | Adam |
| Learning rate | 0.0001 |
